# Training Needs of Community Health Workers Facing the COVID-19 Pandemic in Texas: A Cross-Sectional Study

**DOI:** 10.3389/fpubh.2021.689946

**Published:** 2021-06-14

**Authors:** Courtney Byrd-Williams, Mollie Ewing, E. Lee Rosenthal, Julie Ann St. John, Paige Menking, Floribella Redondo, Stephanie Sieswerda

**Affiliations:** ^1^Health Promotion and Behavioral Sciences, Michael & Susan Dell Center for Healthy Living at The University of Texas Health Science Center at Houston (UTHealth) School of Public Health in Austin, Austin, TX, United States; ^2^Michael & Susan Dell Center for Healthy Living at The University of Texas Health Science Center at Houston (UTHealth) School of Public Health in Austin, Austin, TX, United States; ^3^Department of Medical Education, Paul L. Foster School of Medicine, Texas Tech University Health Science Center, El Paso, TX, United States; ^4^Julia Jones Matthews Department of Public Health, Graduate School of Biomedical Sciences, Texas Tech University Health Sciences Center, Abilene, TX, United States; ^5^University of New Mexico School of Medicine, Albuquerque, NM, United States; ^6^Arizona Community Health Workers Association, Yuma, AZ, United States

**Keywords:** community health worker, workforce training needs, Infectious disease preparedness, COVID-19 pandemic response, education

## Abstract

The COVID-19 pandemic has required the professional healthcare workforce not only to adjust methods of delivering care safely but also act as a trusted sources of information during a time of uncertainty and rapid research and discovery. The Community Health Worker COVID-19 Impact Survey is a cross-sectional study developed to better understand the impact of COVID-19 on this sector of the healthcare workforce, including training needs of those working through the pandemic. The survey was distributed in Texas, New Mexico, and Arizona. This study focuses on Texas, and the data presented (*n* = 693) is a sub-set of qualitative data from the larger survey. Results of the content analysis described in this paper are intended to inform current COVID-19-related CHW training curriculum, in addition to future infectious disease prevention and preparedness response trainings.

## Introduction

The Community Health Worker (CHW) workforce is an integral part of the healthcare system in the U.S. CHWs effectively improve the health of communities through the implementation of community-based public health interventions, especially among underserved populations and communities (1). The COVID-19 pandemic has disproportionately affected communities of color and those who are economically vulnerable (2–4). CHWs may be uniquely placed to help reduce the burden of COVID-19 infections, illness, and death, because they act as a bridge from the communities they serve and the U.S. healthcare system. During the COVID-19 pandemic, there has been a call to prioritize the role of CHWs to help fight the pandemic at the international (5), national (6, 7) and state (8) levels.

In Texas, CHWs can play a pivotal role in combatting the pandemic. Texas is a state with an established CHW workforce (9), and part of the Texas administrative code defines a CHW as “a trusted member, and has a close understanding of, the ethnicity, language, socio-economic status, and life experiences of the community served” (10). Texas CHWs can work as paid employees or volunteers, and according to legislation enacted in 2001, the certification is “mandatory for promotores (as) who are financially compensated for the services that they provide” and “voluntary for promotores (as) who do not receive compensation for their services (11). Texas has a large CHW workforce, consisting of ~4,000 community health workers certified by the Texas Department of State Health Services and an unknown number who are not certified (12, 13).

In order to best equip Texas CHWs in the battle against COVID-19, understanding their training and capacity-building needs related to COVID-19 is critical. To learn about Texas CHWs' training needs, the Maternal and Child Health (MCH) Training Program at UTHealth, a program which provides CEU trainings for CHWs, collaborated with the CHW Core Consensus (C3) Project, a national project that has worked to build consensus to better support the capacity of the CHW workforce to serve individuals and communities (14, 15).

## Materials and Methods

### Study Design

The participants included in the sample for this content analysis were part of a larger cross-sectional survey of CHWs. In the summer of 2020, an interprofessional team of CHWs, CHW instructors, CHW researchers, and maternal and child health researchers developed an online survey to understand the perspectives and experiences of CHWs during the COVID-19 pandemic in Texas, New Mexico, and Arizona. This analysis focuses specifically on a convenience sample of CHWs in Texas. The study was deemed exempt by the Committee for the Protection of Human Subjects (CPHS) at the University of Texas Health Science Center at Houston (HSC-SPH-20-0592). When preparing this manuscript to ensure transparency in reporting the research, the COREQ and STROBE checklists for qualitative and cross-sectional studies, respectively, were used as a guide, where applicable (16, 17).

### Survey Instrument

The questions discussed in this paper are part of the larger survey that aimed to: (1) understand changes in the CHW workforce, (2) identify training opportunities, and (3) describe priority needs of CHWs and their communities. Respondents eligible to participate were people who (1) self-identify as a CHW according to the APHA definition, (2) live in Texas, New Mexico, or Arizona, and (3) have been working as a CHW during and prior to the arrival of COVID-19 in their community. The APHA CHW section defines a CHW as “a frontline public health worker who is a trusted member of and/or has an unusually close understanding of the community served (18).” The survey ended for those who did not meet the inclusion criteria.

Questions used in this analysis were part of a 37-item survey instrument, available in English and Spanish, collecting both qualitative and quantitative responses. First, respondents were asked, “How likely are you to take a free, self-paced online COVID-19 training, specific to CHWs?” Response options were a five-point Likert scale ranging from “very unlikely” to “very likely.” Second, respondents who answered “likely” or “very likely,” received a follow-up question to elicit training topics of interest in a free-text answer box: “What topics related to COVID-19 would be most helpful to you as a CHW?”

Data collection utilized Qualtrics XM online survey software. All survey instructions, questions, and answers were available to respondents in Spanish or English. The first set of instructions, prior to the screening questions, included guidance on how to toggle between Spanish and English at any point in the survey. The survey content went through three translation phases: (1) the “translate survey” feature in Qualtrics, (2) feedback and edits from survey team members fluent in Spanish, and (3) an additional round of feedback and edits from Spanish-speaking team members. To ensure optimal user-experience, functionality, readability, and survey length, the survey content was piloted by English- and Spanish-speaking students at UTHealth School of Public Health.

### Survey Distribution

Local and state-level CHW associations and organizations collaborated with the survey team to distribute the survey link. Survey distribution occurred via email with the access link—avoiding social media platforms to decrease risk of fraudulent responses (19). To maximize the survey's reach, the survey team engaged 15 community partners at local and state-level CHW organizations and associations in the months leading up to data collection.

Distribution collaborators received an optional email template for convenient marketing of the survey, that included the survey hyperlink and the survey URL. Additionally, the survey link was distributed via professional contacts of the survey team. The exact number of people who received the survey is indeterminable due to the phone-tree style of distribution and the potential of a single CHW receiving the survey link from multiple distributing sources. At the end of the survey, respondents were given the opportunity to provide their email address to be included in a give-away of ten $100 e-gift cards to a large retail chain with over 500 stores in Texas.

### Data Collection

Data collection occurred July 13 to August 3, 2020, with the original completion date of July 27, 2020. A one-week extension attempted to reach additional CHWs. Distributing collaborators received marketing materials in June of 2020, on the survey go-live date, and about 2 weeks after the go-live date with information of the 1-week extension. Documentation of the actual dates that distribution collaborators completed outreach to their listservs was not collected. After data collection, the research team prepared and shared a report with stakeholders, including survey respondents and organizations that collaborated in distributing the survey (20). The secure network at UT Health School of Public Health houses the data.

### Data Analysis

First, descriptive frequencies were conducted in SPSS for demographic data and the question of how likely respondents would be to take “a free, self-paced online COVID-19 training, specific to CHWs.” Second, respondents who indicated they were they were “likely” or “very likely” to take a training were asked, “What topics related to COVID-19 would be most helpful to you as a CHW?,” and a content analysis was conducted to categorize the open-ended, de-identified survey responses from the free text entry question (21).

To systematically sort, synthesize, and summarize the free text responses into training topic categories, two researchers (ME & SS) conducted an inductive-dominant content analysis (22), with a third reviewer who resolved discrepancies (CBW). There were no a priori categories, and categories emerged from the data. In an iterative process, the researchers created and refined the categories and sub-categories of training topics and created a codebook to help ensure an accurate and objective categorization process. Inclusion criteria for each category and sub-category were identified utilizing words and phrases, which were taken verbatim from survey respondent's answers. Some respondents provided more than one training topic, and each response was classified into as many categories as needed. After several iterations of data reduction and category creation, a last review was conducted using the finalized codebook. No new category or sub-category was identified during the final analysis.

## Results

### Demographics and Training Interest

Demographic characteristics of the respondents included in this content analysis (*n* = 693) are presented in Table 1. Nearly 90% of survey respondents are female, 65% of Hispanic, Latino, or Spanish origin, 18% Black or African American, 64% White, 94% are certified in Texas as CHWs, and 16% completed the survey in Spanish, and their average age is 47 years (SD = 10.8). After the survey screening questions, 885 respondents agreed to the consent. Of the 885 eligible respondents from Texas, 801 answered the question about how likely they would be to take a self-paced, online, COVID-related training. Of those, 747 (93%) responded that they would be “likely” or “very likely” to take a training.

**Table 1:** Demographic characteristics of Community Health Worker (CHWs) respondents who indicated that they were “Very Likely” or “Likely” to take a free, self-paced online COVID-19 training specific to CHWs and provided a response to the following question: “What topics related to COVID-19 would be most helpful to you as a CHW?” (*n* = 693).

**Characteristics**	***n(%)***
**Age in years [Mean (SD)], (*****n*** **=** **687)**	47.67 (10.82)
**Number of years working as CHW [Mean (SD)], (*****n*** **=** **682)**	7.22 (5.85)
**Percentage who completed survey in Spanish (*****n*** **=** **693)**	108 (15.58)
**Percentage certified as CHW in Texas**	652 (94)
**Sex (*****n*** **=** **692)**	
• Male	70 (10.12%)
• Female	618 (89.31%)
• Other	1 (0.14%)
• Prefer not to say	3 (0.43%)
**Ethnicity (*****n*** **=** **692)**	
• Hispanic or Latino	454 (65.61%)
• Not Hispanic or Latino	232 (33.53%)
• Prefer not to say	6 (0.87%)
**Race (*****n*** **=** **681)**	
• White	438 (64.32%)
• Black or African American	129 (18.94%)
• American Indian or Alaska Native	5 (0.73%)
• Asian	22 (3.23%)
• Other	63 (9.25%)
• Multiracial	24 (3.52%)
**Likelihood to take a free, self-paced online COVID-19 training specific to CHWs (*****n*** **=** **693)**	
• Very Likely	584 (84.26%)
• Likely	109 (15.73%)

### Categorization of Training Topics

Of the 747 respondents that expressed interest in training, 93% (*n* = 693) provided training topics of interest in the free text answer box. Respondents provided from one (*n* = 483) to five topics (*n* = 3) for a total of 970 topics from 693 respondents, with a median of one topic per respondent. Many gave broad responses wanting “any” information on COVID-19, while others provided more specific and detailed topics. Categories and sub-categories along with example text are presented in Table 2 and described below. A total of seven categories and 22 sub-categories were identified. An exhaustive list of key words used to categorize the categories and sub-categories and select quotes from CHW respondents are included in Appendix 1. The seven major topics and related sub-categories are described below.

**Table 2:** Categories & sub-categories of training topics related to COVID-19 requested by Community Health Workers (CHWs).

**Category (*n*) ** • Sub-category	**Example text ** (examples text from CHW responses for each training topic category and sub-category)
**Prevention (*****n*** **=** **289)**	
• COVID-19 Prevention (General)	Hygiene, cleaning/disinfection, quarantine, isolation after exposure, transmission
• Masks/PPE/Social Distancing	Facial coverings, personal protective equipment, social distancing
• Vaccine	Vaccination, immunization, vaccine
• Of illness through Health/Wellness	Promoting health/wellness and regular health care, importance of diet & exercise
• Contact Tracing	Contact tracing, tracking cases, tracking, tracing
**Clinical course of COVID-19 (*****n*** **=** **188)**	
• Treatment	Treatment, managing symptoms, curing COVID-19
• Symptoms	What COVID-19 does to the body, signs and symptoms, asymptomatic
• Testing	Tests, where to get tests, testing sites, accuracy of tests, testing costs
• Post-COVID Impact and Care	After effects, after care, antibodies, reinfection
**Community resources and engagement (*****n*** **=** **103)**	
• General resources	Resources, community services
• Specific resources	Transportation, medicine, rental assistance, food assistance, evictions, financial help
• Communication	Engaging clients, how to talk to the community, outreach, how to provide info effectively
• How to work remotely as a CHW	Delivering information virtually, helping clients remotely
**Vulnerable populations (*****n*** **=** **102)**	
• People at increased risk^a^	Older adults, “co-morbidities,” diabetes, immunocompromised/HIV-positive
• Others who need extra precautions^b^	Rural communities, people with disabilities, breastfeeding parents, refugee populations
• Children/kids	Children/kids, schools, effects of COVID-19 on children
**Mental Health (*****n*** **=** **79)**	
• General	Coping, depression, anxiety, stress/stress management
• Of health care providers	CHW self-care, compassion fatigue, mental and emotional health for health workers
• Due to isolation	Isolation, loneliness, how to treat anxiety generated by confinement
• Due to dealing with illness	Coping if client for family member has the virus, how to deal with loss of a loved one
**General COVID-related information (*****n*** **=** **118)**	
• General COVID-related information	All/anything and everything, myths, education and information
**Other/unclear (*****n*** **=** **91)**	
• Other	Appeared ≤ 3 times in the responses, e.g., motivational interviewing, hurricanes
• Unclear	Text was unclear, i.e., not clear to any of the researchers or interpreted differently

a *Groups at increased risk for severe illness of COVID-19 (https://www.cdc.gov/coronavirus/2019-ncov/need-extra-precautions/index.html)*.

b *Those who might need to take extra precautions against COVID-19 (https://www.cdc.gov/coronavirus/2019-ncov/need-extra-precautions/other-at-risk-populations.html)*.

#### Prevention

This topic category was the most frequently reported and more than a third of respondents requested a training topic related to the prevention of COVID-19. This category included the four sub-categories of “General prevention,” “Masks/personal protective equipment (PPE) social distancing,” “Vaccines,” and “Health/wellness promotion”.

The sub-category of “General prevention” was the most frequently reported and included responses such as “prevention,” “disinfecting,” and “hygiene, safety.” Less frequently reported were the sub-categories of “Masks/PPE/Social Distancing,” “Vaccines,” and “Health/wellness promotion.”

The “Masks/PPE/Social Distancing” sub-category was created, because respondents frequently grouped them, e.g., “the importance of social distancing and use of facial masks” and “social distancing, importance of the mask.” The respondents discussed PPE for the community, “where to get free PPE for the community” and “PPE needs for the public,” but not specifically in a clinical setting.

The sub-categories of “Vaccine,” “Health/wellness promotion,” and “Contact tracing” had fewer than 25 responses. “Vaccine” included straightforward responses, e.g., “vaccine,” “vaccines that are discovered.” The sub-category of “Health/wellness promotion” had responses that included the “importance of diet and exercise” and the “promotion of regular healthcare.” The “Contact Tracing” sub-category included straightforward responses such as “contact tracing,” “tracking cases,” and “tracing.”

#### Clinical Course of COVID-19

This category includes all sub-categories related to the clinical course of COVID-19, including “Symptoms,” “Testing,” “Treatment,” and “Post-COVID impact and care”.

Among these sub-categories, the sub-category of “Treatment” was the most frequently reported (*n* = 58) and included responses such as “treating symptoms,” “how to manage symptoms at home, and “curing COVID-19.” “Symptoms” and “Testing” were the next most frequently reported in this category, with 54 and 49 responses, respectively. “Symptoms” included many straightforward responses such as “symptoms” and “signs and symptoms”, but also responses such as “what COVID-19 does to the body” and “pathophysiology of COVID-19.” The “Testing” sub-category included responses that indicated CHWs would like more information about “accuracy of tests” and “how often to get tested,” and information about testing resources, such as “where to get tested,” “promoting testing,” and “testing costs.”

The “Post-COVID Impact and Care” sub-category (*n* = 27) was created because respondents often reported a desire to know more about the “after-effects of COVID-19” or the impact of “COVID-19 antibodies” on reinfection and treatment of the disease.

#### Community Resources and Engagement

About 50 respondents indicated that they would like a training on “Community Resources,” which was divided into two sub-categories, i.e., general and specific. Some respondents broadly requested a training on “resources” or “community services.” Other respondents requested education on more specific community resources, e.g., “food, medication, utilities, etc.” and “assistance with rent.” Another 50 respondents requested training in “Community Engagement,” which was divided into two sub-categories, i.e., “Communication” and “Working remotely as a CHW.” The sub-category ‘Communication' included responses such as “community engagement” and “how to talk to the community about COVID.” The sub-category “Working remotely as a CHW” included responses such as “how to reach goals serving families while working from home.”

#### Vulnerable Populations

Over 100 respondents included responses related to “Vulnerable Populations,” or the specific community or populations they serve. The three sub-categories that make up this category include “People at Increased Risk,” “Other People Who Need Special Precautions,” and “Children/Kids.” The titles and definitions of these sub-categories reflect the CDC guidance around people who are known to be at increased risk for severe illness due to COVID-19 or those who may be at increased risk and need special precautions (CDC).

The “People at Increased Risk” sub-category (*n* = 46) includes groups such as “older adults,” “those with co-morbidities,” “those who are pregnant,” and “immunocompromised/HIV-positive individuals.” The “Other People Who Need Special Precautions” sub-category (*n* = 31) includes groups who *may* be at increased risk, such as “refugee populations”, “people experiencing homelessness,” and “racial & ethnic minority groups.” Responses related to “Children/Kids” made up about one-quarter of this category's responses (*n* = 25), and included responses such as “how to care for children with COVID-19,” “schools,” “child care settings,” and the “effects of COVID-19 on children.”

#### Mental Health

Similar to “Community resources,” about 80 respondents indicated they would like a training on “Mental health,” which was divided into sub-categories, i.e., “General mental health,” “Mental health of healthcare providers,” “Mental health due to isolation,” and “Mental health due to dealing with personal/family illness.”

#### General COVID-19-Related Information

Over 10% of respondents gave a very broad response indicating they would like a COVID-19-related training on “anything” and “everything” or about “COVID facts” and “myths.” Respondents who reported they would like a training about “education” or “information” were also sorted into this category.

#### Other/Unclear

*Seven* responses fell into the “Other” category, which was comprised of topics that appeared <5 times, including “employee rights” (*n* = 3), “intimate partner violence” (*n* = 2), “motivational interviewing certification” (*n* = 1), and “COVID during hurricanes” (*n* = 1). Over 80 responses were unclear to the researchers conducting the content analysis, meaning that the responses were too vague or confusing to develop COVID-19-related training/learning objectives. Unclear responses included text such as “what is really going on?” and “cancer prevention.”

## Discussion

This study describes priority training needs and interests during the COVID-19 pandemic reported by CHWs in Texas. While some studies have compiled guidelines and resources for CHWs during COVID- 19 (23), this is the first study to our knowledge that provides recommendations for training topics requested by CHWs in Texas. Recognizing the professional changes and training needs of CHWs creates the opportunity to support this sector of the healthcare workforce during the current pandemic and to prepare for future public health emergencies. The categories identified by the respondents that could be helpful in developing CHW trainings included how to prevent COVID-19 (“Prevention”); the diagnosis, treatment, and effects of the illness (“Clinical Course of COVID-19”); the effects of COVID-19 on specific populations who are vulnerable to the disease (“Vulnerable Populations”); the effects of COVID-19 on mental health (“Mental Health”); and how to engage and provide resources to the community (“Community Resources and Engagement”).

The categories and sub-categories presented from this study can serve as a starting point for those developing trainings. To ensure training relevance, the research team recommends collaborating closely with CHWs themselves to identify specific learning objectives and co-create training content. This tailoring is especially important given the fluctuating nature of the pandemic and our response. As infection rates and state/local restrictions change, so will training needs.

In addition to the categories discussed above, a “General COVID-related information” category emerged from the data. This category is not intended to provide guidance on training content, but it does provide some insight. Given that over 10% of respondents indicated that they were interested in “any” or “all” topics related to COVID-19 demonstrates, it appears that there is a need and hunger for COVID-related trainings among CHWs. However, at this point in the pandemic CHWs may have more specific questions and training needs. An important consideration for any of the training topics presented in this paper is the rapidly changing rates of COVID-19 infections, hospitalizations, and deaths during the time of data collection and the publishing of this article.

The list of training categories that emerged from the data in this study is most certainly not an exhaustive list of COVID-19-related training topics. Of the 970 topics provided by respondents, only seven were categorized into the “Other” category, meaning the topic was mentioned by fewer than three respondents and not easily incorporated into an existing category. The unassigned topics included (1) motivational interviewing, (2) intimate partner violence, (3) COVID-19 and hurricanes, and (4) employee rights and employer responsibility during COVID-19. Though these topics were not frequently requested, they are relevant. For example, studies have shown that CHWs are effective at using motivational interviewing (MI) to elicit behavior change (24), and MI could be used to help clients practice safe pandemic behaviors. Second, some studies suggest an increase in intimate partner violence (IPV) as a consequence of COVID-19 related lock-down restrictions, sheltering-at-home, isolation, and quarantine (25), and as frontline workers working with families, CHWs are well-positioned to help prevent IPV (26). Third, as the pandemic continues, it is important to consider COVID-19 recommendations in disaster preparedness plans, such as those for hurricanes, to mitigate negative health outcomes if these challenges were to converge (27). Finally, several CHWs requested training topics related to employee rights and employer responsibilities during the pandemic, i.e., sick days and protective equipment. Future studies should examine whether CHWs felt protected or whether they felt their employee rights were violated while working during the pandemic.

The most frequently requested training category was “Prevention.” Some responses were broad and requested training topics on how to “prevent COVID” or “stop the spread,” while others responses fell into more specific sub-categories. One sub-category was “Masks/PPE/Social Distancing,” and CHWs, who are trusted in the community, could provide information to people on the ever evolving COVID-19-related information and recommendations from the CDC. “Vaccines” were another sub-category of training topics that was identified, and at the time of data collection, no COVID-19 vaccines had been authorized for emergency use in the U.S. by the Food and Drug Administration. At the time of publishing, at least three vaccines have been authorized for emergency use (28), so the training requests related to vaccines may have changed. Other prevention related topics were related to “Health and Wellness,” such as encouraging regular health care, presumably because people have been delaying regular health care, such as cancer screening exams, during the pandemic (29).

CHWs requested trainings to help their communities by improving access to community resources and increasing community engagement. Specific community resources that were requested aligned with the social determinants of health, including food availability, employment, housing, and access to healthcare. The COVID-19 pandemic has increased the number of households experiencing food insecurity (30), impacting vulnerable populations (31). Given their foothold in vulnerable communities and their resourcefulness, CHWs could provide the link between community members and the resources they need. CHWs also requested trainings on how to engage the community, specifically while working remotely. A recent study that conducted focus groups with CHWs about their experiences during the pandemic identified a similar theme of technology and the need and opportunity to develop skills (32).

### Recommendations for Those Who Develop Trainings or Training Courses

The training topics identified in this study are intended to serve as guidance. Categories and sub-categories were developed through the lens of training development opportunities and which categories and sub-categories could be used to develop learning objectives. Topics identified through this survey cross a wide range. We see several topics as having an enduring value to sustained training opportunities and competency development for CHWs related to public health preparedness overall. Notably, the topic of prevention, most commonly cited by participants, matches the recommendations of the C3 Project for core skill competency area that falls within the Project's core competency Knowledge-base area (https://www.c3project.org/resources). The Knowledge-base area has expanded significantly over the 20 years since the original release of core CHW roles and competency recommendations in the National Community Health Advisor Study in 1998 (33).

### Training and Capacity Building for CHW Preparedness

The COVID-19 pandemic has created a learning laboratory for CHWs and other community-centered health care workers as they have become essential partners in many regional efforts to do contact tracing and promote healthy behaviors to combat the spread of virus (34). Long-range training and capacity–building plans for CHWs moving forward from COVID-19 should emphasize prevention within a preparedness framework. A national project carried out by the Association of Schools of Public Health (ASPH) at the request of the Centers for Disease Control and Prevention (CDC) in response to the Pandemic and All-Hazards Preparedness Act of December 2006 (35) led to the development of preparedness public health core competencies. Four core areas of the competency map are: Model Leadership, Communicate and Management of Information; Plan for Improved Practice; and Protect Worker Health and Safety. Areas that emerged from our data include some of these core elements. Future exploration of overlap and divergence is recommended.

### Training Characteristics to Promote Capacity-Building

In the pandemic, it is clear that online training has now been normalized and it must be considered along with all other training modalities. Based on previous interviews with CHWs, there is a preference for short, self-paced, online courses, especially for continuing education units (CEUs) needed to maintain certification. While this type of training is often more accessible, it is not as effective at meeting learning objectives as higher intensity trainings (1). While some CHWs may prefer brief, self-paced, online courses, other CHWs will prefer synchronous and interactive trainings offered via an online videoconferencing platform or in-person, though these have not been a norm during the COVID-19 pandemic. Additionally, online courses may not be accessible to the ~1-third of rural Texans that have limited access to high-speed internet and communities along the US/Mexico border that rank among the nation's “worst-connected” cities (36).

In addition to approaches to training, it is also vital to consider language access when planning trainings for and with CHWs. In this study, 16% of survey respondents answered in Spanish. Over a third of Texans over the age of five speak a language other than English at home (37). After Spanish, the most frequently spoken languages are Vietnamese and Chinese with more than 300,000 people speaking those languages (37).

### CHWs as Trainers- Building Capacity of the CHW Field From Within

It is important to consider whose expertise is emphasized when trainings are developed. As recommended by the CHW National Education Collaborative, when considering how to approach CHW training, CHWs should play a prominent role in facilitating educational sessions (38). One example of CHW-led training during the COVID-19 response efforts comes from the Arizona Community Health Worker Association (AzCHOW).

The CHW leadership team of AzCHOW has been offering virtual sessions in Spanish for other CHWs to both offer social support and to promote skill development so CHWs may better provide needed support to their communities (39). Most recently, the online sessions have focused on vaccine hesitancy and access to vaccines for both CHWs and their communities. We observe that CHW–led training by and for CHWs during the pandemic exemplifies the CHW–skill building learning laboratory created by the pandemic noted earlier.

### Limitations

It is important to note several limitations of this study. First, the timing of the study may limit the generalizability. As discussed previously, the changing nature of the pandemic could impact the training topics that are requested. Data represent responses reported earlier on in the pandemic (July-August 2020), and responses may not reflect current CHWs' needs and opinions now a year into the pandemic. Additionally, there were at least three other surveys of CHWs circulating around the time of data collection for this study, which could have increased survey fatigue among CHWs and lowered the response rate for this study. Also, the survey asked CHWs to provide topics for a free, self-paced, online training, because that is the type of training the MCH Training Program has previously developed for CHW CEUs. The survey did not ask about other training formats or modalities that respondents may be interested in and, as such, the results from this study may not be generalizable to other types of trainings. Lastly, the survey did not include questions about how applicable the trainings would be to certification or the maintenance of certification, which would provide insight into CHWs motivations for completing trainings. Future research should explore the motivations of both certified and non-certified CHWs to inform the development of trainings.

### Recommendations for Researchers

Survey respondents yielded a large volume of qualitative data when given the opportunity to complete free text answers. Future researchers studying the CHW population are encouraged to consider (1) free text response as an effective method of eliciting data and (2) plan for bandwidth and expertise available during the qualitative data analysis phase. To elicit more specific training needs and avoid misinterpretation in future survey instruments, researchers recommend providing general training topics, such as the themes identified in this paper, for respondents to select prior to then providing an open text answer for respondents to identify specific training needs.

In reflecting on the survey process undertaken, we add that working directly with significant CHW input in the design, delivery, and interpretation of research tools such as surveys is best practice (40). This team included a key CHW member but haste due to COVID-19 impacted our further pursuit of greater input. Future work in this arena on an upcoming follow-up survey will include direct input from CHWs through several mechanism including town hall and an all-CHW advisory group.

## Conclusions

Community Health Workers role in preparedness has not been well-defined, though it has been examined in other public health emergencies (41). The COVID-19 pandemic has brought new attention the field of public health's role in preparedness overall and with it, new attention to public health's frontline team members, CHWs, who are increasingly identified as essential to address the pandemic and health equity and access issues overall. This paper examines preparing CHWs for their emerging preparedness role in the wake of the COVID-19 Pandemic. We ask, what might this enhanced role of CHWs in preparedness mean for CHWs going forward? We call for future investigation grounded in a community–based participatory research framework to help move this dialogue forward while fully engaging CHW leadership nationally and internationally. Through this engaged action research approach, CHWs can work to define their terms for any preparedness roles they will play in the future and any related capacity building they will require to fulfill those roles in the face of inevitable global challenges ahead.

## Data Availability Statement

The raw data supporting the conclusions of this article will be made available by the authors, without undue reservation.

## Ethics Statement

The studies involving human participants were reviewed and approved by Committee for the Protection of Human Subjects (CPHS) at the University of Texas Health Science Center at Houston (HSC-SPH-20-0592). The patients/participants provided their written informed consent to participate in this study.

## Author Contributions

All authors contributed to the conception of the study design, the acquisition and interpretation of the data, and manuscript drafting. SS, ME, and CB-W conducted the data analysis. All authors approved the final version and agree to be accountable for all aspects of the work. A report from this survey, which did not include this content analysis, is available online at: https://sph.uth.edu/research/centers/dell/resources/the%20final%20report%20of%20the%20community%20health%20worker.pdf.

## Conflict of Interest

The authors declare that the research was conducted in the absence of any commercial or financial relationships that could be construed as a potential conflict of interest.
