# Supplementary material for: Training Needs of Community Health Workers Facing the COVID-19 Pandemic in Texas: A Cross-Sectional Study
Source: Front Public Health. 2021 Jun 14;9:689946. doi: 10.3389/fpubh.2021.689946 (PMC8236534; doi:10.3389/fpubh.2021.689946)
Supplement: Supplementary file 1 [file Table_1.DOCX]

**APPENDIX**

**Appendix 1**: Inclusion criteria for themes and training topics related to COVID-19 most frequently requested by CHWs who indicated they were “Very Likely” or “Likely” to take a free, self-paced online COVID-19 training specific to CHWs and provided a response to the following question: “What topics related to COVID-19 would be most helpful to you as a CHW?” (n = 693)

| **Category (n)**   - Subcategory | **Key words used to categorize** | | **Example excerpts of text from CHWs** |
| --- | --- | --- | --- |
| **Prevention (n = 289)** | | | |
| - COVID-19 Prevention (General) | - Stop spread - Precautions - Hygiene - Cleaning/disinfecting - Risk reduction/mitigation - Protect from exposure - Precautionary measures | - Health and safety - Keeping the population safe - Safety measures - Security - Personal safety - Screening - Isolation after exposure - Quarantine - Transmission | “Health and safety regarding respiratory diseases, Standard precautions: Cleaning and disinfection” |
| - Masks/PPE/Social Distancing | - How to tell others to wear masks - Effectiveness of masks - Importance of masks - How to teach others to wear masks - Social distancing - How to properly put on PPE - Protective wear | | “the importance of social distancing and use of facial masks”  “how to properly put on, wear, and take off PPE” |
| - Vaccine | - Vaccination - Immunization - Vaccine | | “If there will be a vaccine soon.” |
| - Health/Wellness Promotion; Diet/Nutrition | - Promoting health and wellness - Health management - Promoting regular healthcare - Importance of diet and exercise - Way to manage health - Healthy habits | | “Promoting Health & Wellness and Resources”  “Ways to manage health, importance of taking medications, monitoring health” |
| - Contact Tracing | - Contact tracing - Tracking cases - Tracking - Tracing | | “Contact tracing”  “Tracking” |
| **Clinical Course of COVID-19 (n = 188)** | | | |
| - Treatment | - Treatment - Treating symptoms - Managing symptoms - How to manage symptoms at home - Curing COVID-19 | | “tips for people on how to manage symptoms at home”  “How to help people who have been diagnosed with COVID”  “Care of COVID-19 person at home. Treating the symptoms.” |
| - Symptoms | Symptoms  What covid-19 does to body  Signs and symptoms  Asymptomatic | | “signs and symptoms for age groups, races, and genders”  “Asymptomatic people” |
| - Testing | Tests  Testing  Where to get tested  Assist with testing  Testing sites  Promoting testing  Accuracy of tests  How often to get tested  Testing costs  Testing procedures | | “explaining the differences in test, pcr/antibody--"  “where to get tested”  “accuracy of tests” |
| - Post-COVID Impact and Care | After effects  After care  Antibodies  Reinfection | | “What Are The After Affects”  “After care”  “something along the lines of antibodies and if that should help with not getting covid 19 again.” |
| **Community Resources and Engagement (n = 103)** | | | |
| - General Resources | Resources  Community services | | “resource assistance”  “community resources” |
| - Specific Resources | Transportation  Medicine  Rent assistance  Food assistance  Utility assistance  Help with evictions  Financial help  Resources for those who have lost employment  Handouts/educational tools | | “helping people facing evictions”  “Financial Help during Pandemic”  “Community Services available, immunizations, clinics, shelters, supplies, transportation”  “How to Access Support/food/assistance with Rent” |
| - Communication | Engaging clients  How to talk to the community  Outreach  How to provide info effectively  Reaching the community | | “How to talk to the community about COVID, hygiene, risks, responsibilities”  “outreach in the community” |
| - How to Work Remotely as a CHW | Delivering information virtually  Helping clients remotely | | “How to reach goals serving families while working from home.”  “New ways to help with behavioral changes remotely” |
| **Vulnerable Populations (n = 102)** | | | |
| - People at Increased Risk^[[1]](#footnote-1)^ | Older adults  “co-morbidities”  Diabetes  Immunocompromised/hiv-positive  Pregnancy  Obesity  Asthma  Neurologic conditions, such as dementia (n=1) | | “Diabetes and Covid, At risk age groups”  “Underlying conditions of people that are high risk.”  “Chronic diseases and obesity with Covid-19” |
| - Other People Who Need Extra Precautions^[[2]](#footnote-2)^ | Rural communities  People with disabilities  Developmental and behavioral disorders  Breastfeeding  Nursing homes/long term care facilities  Caregivers of people living with dementia  Refugee populations  Drug use and substance use disorder  People experiencing homelessness  Racial & ethnic minority groups  Limited-English-proficient populations  Low-Income Individuals/Poverty | | “COVID-19 effects on those with low health literacy/ Attitudes of refugees who have been through trauma prior to CV19 (is COVID-19 impacting them further or just something else they have to 'go through'?) COVID-19 and its impact on wellness and sick care”  “Homelessness”  “COVID-19 and Implications to Native Americans, African Americans & Refugee Populations” |
| - Children/Kids | Children/kids  How to care for children with COVID  Schools  Child care settings  Effects of COVID-19 on children | | “How to care for children with COVID-19 how to protect the children from COVID-19 if they are too young to wear a mask.”  “What is the upcoming learning environment in schools?” |
| **Mental Health (n = 79)** | | | |
| - Mental Health (General) | Mental health issues due to COVID-19  Coping  Therapy services  Depression  Anxiety  Stress/stress management  Behavioral health  Well being | | “Anxiety and depression related to COVID19”  “Mental Health and Coping During COVID-19”  “How to handle the stress during a pandemic” |
| - Mental Health of Health Care Providers | CHW self-care  Mental and emotional health for health workers  Compassion fatigue  Self-care/burnout | | “Selfcare of a CHW”  “Mental Health and Trauma Training (Compassion Fatigue or Self-Care)” |
| - Mental Health due to Isolation | Isolation  Loneliness  How to treat anxiety generated by confinement  Addressing social needs | | “The mental health aspect of dealing with isolation”  “how to support clients why they are on isolation/quarantine precautions” |
| - Mental Health due to Dealing with Personal/Family Illness | Coping if client or family member has the virus  How to deal with the loss of a loved one | | “coping skills for clients that have virus”  “How to deal with family who have lost a person to Covid-19” |
| **General COVID-related Information (n = 118)** | | | |
| - General COVID-related Information | All/anything and everything  Myths  Education and information | | “Everything there is to know”  “All issues related to Covid since I need the official and truthful information to be able to help my communities more” |
| **Other/Unclear (n = 91)** | | | |
| - Other/Unclear | Unclear/vague responses, not sure what learning objectives would be for the topic  Small number of responses, clear on what the learning objectives would be for the topic | | “What is really going on?” (unclear)  “cancer prevention” (unclear)  “the rights an employee has in the face of COVID-19” (other) |

1. https://www.cdc.gov/coronavirus/2019-ncov/need-extra-precautions/people-with-medical-conditions.html?CDC_AA_refVal=https%3A%2F%2Fwww.cdc.gov%2Fcoronavirus%2F2019-ncov%2Fneed-extra-precautions%2Fgroups-at-higher-risk.html [↑](#footnote-ref-1)
2. https://www.cdc.gov/coronavirus/2019-ncov/need-extra-precautions/other-at-risk-populations.html [↑](#footnote-ref-2)
